# Interaction of Salmonella enterica Serovar Typhimurium with Intestinal Organoids Derived from Human Induced Pluripotent Stem Cells

**DOI:** 10.1128/IAI.00161-15

**Published:** 2015-06-15

**Authors:** Jessica L. Forbester, David Goulding, Ludovic Vallier, Nicholas Hannan, Christine Hale, Derek Pickard, Subhankar Mukhopadhyay, Gordon Dougan

**Affiliations:** a Wellcome Trust Sanger Institute, Wellcome Trust Genome Campus, Hinxton, United Kingdom; b Wellcome Trust-Medical Research Council Stem Cell Institute, Anne McLaren Laboratory, Department of Surgery, West Forvie Site, University of Cambridge, Cambridge, United Kingdom

## Abstract

The intestinal mucosa forms the first line of defense against infections mediated by enteric pathogens such as salmonellae. Here we exploited intestinal “organoids” (iHOs) generated from human induced pluripotent stem cells (hIPSCs) to explore the interaction of Salmonella enterica serovar Typhimurium with iHOs. Imaging and RNA sequencing were used to analyze these interactions, and clear changes in transcriptional signatures were detected, including altered patterns of cytokine expression after the exposure of iHOs to bacteria. *S*. Typhimurium microinjected into the lumen of iHOs was able to invade the epithelial barrier, with many bacteria residing within Salmonella-containing vacuoles. An *S*. Typhimurium *invA* mutant defective in the Salmonella pathogenicity island 1 invasion apparatus was less capable of invading the iHO epithelium. Hence, we provide evidence that hIPSC-derived organoids are a promising model of the intestinal epithelium for assessing interactions with enteric pathogens.

## INTRODUCTION

Salmonellae are enteric bacterial pathogens that can interact with, and have the capacity to invade, the intestinal mucosal surface (1). Globally, salmonellae constitute a huge disease burden, with over 90 million gastroenteritis and 22 million typhoid cases occurring per year (2). Characterization of the early interactions with the human epithelial response could provide significant insight into how salmonellae cause disease (3). Control of infection is likely achieved through multiple mechanisms, including cytokine signaling and secretion, inflammasome activation, production of reactive oxygen species and antimicrobial peptides, and phagocyte-mediated microbial killing (4–6).

Human-based *in vivo* systems are confounded by the technical challenges of quantifying the interactions between a pathogen and a mucosal surface, which are likely to be rapid and dynamic (7). The use of model organisms such as mice to study salmonellae is commonplace; however, the diseases caused by Salmonella enterica serovar Typhimurium differ between mice and humans. In the mouse, *S*. Typhimurium spreads systemically, and this is characterized by colonization of the liver and spleen (8), whereas *S*. Typhimurium causes predominantly localized gastroenteritis in healthy humans. Therefore, the development of alternative human *in vitro* models for the study of *S*. Typhimurium is desirable. Studies on Salmonella interaction with the human intestinal epithelium have been further hindered by human cell culture systems (9), which lack the three-dimensional architecture (10) and different cell types that make up the intestinal epithelium. Thus, it would be desirable to have alternative models in which to study these important human pathogens.

Intestinal human organoids (iHOs) are a multicellular, human-specific system that can be used to study host-pathogen interactions at the intestinal interface (11–13). iHOs harbor a mixture of cell types normally present in the intestinal epithelial barrier *in vivo*, including goblet cells, Paneth cells, enterocytes, and enteroendocrine cells (14). iHOs can be derived from human induced pluripotent stem cells (hIPSCs) (14), which are adult somatic cells that have been reprogrammed to pluripotency by the forced expression of a set of reprogramming factors (15). iHOs have been exploited to study several different pathogens, including Clostridium difficile (16) and rotavirus (12). Characterization in response to infection of mouse intestinal organoids derived from adult primary stem cells (17) has also been investigated (11, 13), demonstrating they can secrete functional antimicrobial peptides and that salmonellae disrupt tight junctions and activate inflammatory responses.

Here, we investigated the utility of iHOs as a model of *S*. Typhimurium infection and define the baseline interactions by using both wild-type and mutant bacteria. We conclude that iHOs provide a useful system for interrogating some of the complex early interactions between the enteric pathogen *S*. Typhimurium and the intestinal epithelium of the human host.

## MATERIALS AND METHODS

### Bacterial strains and culture conditions.

*S*. Typhimurium SL1344, a well-characterized isolate used routinely in laboratory experiments (18), was used in these studies. In order to examine iHOs during an attenuated bacterial infection, we used an *S*. Typhimurium SL1344 strain defective in the *invA* gene of Salmonella pathogenicity island 1 (SPI-1). *S*. Typhimurium SL1344 carrying an *invA* deletion was constructed by moving the *invA*::Km^r^ deletion from *S*. Typhimurium SL3261 to SL1344 by P22 transduction. *invA* mutant *S*. Typhimurium has been described previously (19). Bacteria were grown in Luria-Bertani broth overnight at 37°C with shaking. Before infection, bacterial cells were resuspended at 2 × 10^7^ CFU ml^−1^ in Dulbecco's phosphate-buffered saline (Sigma-Aldrich), with 1 × 10^6^ bacteria added per well of a 24-well plate of organoids. For microinjection, bacterial cultures were diluted 1:1 with phenol red (Sigma-Aldrich).

### hIPSC culture.

hIPSCs were grown under feeder-free conditions as previously described (20). To allow adherence of hIPSC colonies, tissue culture plastic (Corning) was precoated with porcine gelatin (1 g/liter; Sigma-Aldrich) dissolved in water for embryo transfer (Sigma-Aldrich) for 30 min, followed by MEF medium consisting of Dulbecco's modified Eagle's medium (DMEM; Invitrogen), 10% fetal bovine serum (Biosera), 1% 200 mM l-glutamine (Invitrogen), and 1% penicillin-streptomycin (10,000 U ml^−1^; Invitrogen). hIPSCs were maintained feeder free at 37°C and 5% CO_2_ in chemically defined, serum-free IPSC maintenance medium (CDM-PVA) consisting of 50% DMEM/F-12 (Invitrogen), 50% Iscove's modified Dulbecco's medium (Invitrogen), 5 ml of chemically defined lipid concentrate (Invitrogen), 450 μM monothioglycerol (Sigma), 7 μg ml^−1^ insulin (Roche), 15 μg ml^−1^ transferrin (Roche), 1 mg ml^−1^ PVA (Sigma), and 10,000 U ml^−1^ penicillin-streptomycin (Invitrogen) supplemented with activin A (10 ng ml^−1^; R&D Systems) and basic fibroblast growth factor (bFGF, 12 ng ml^−1^; R&D Systems). The medium was changed daily, and hIPSCs were passaged by using a mixture of collagenase and dispase at a ratio of 1:1 every 5 days. The hIPSC line used in this study was A1ATD-1 (21).

### Growth and passaging of intestinal organoids.

Before beginning differentiation, hIPSCs were passaged and grown for 2 days (days 0 and 1) under CDM-PVA maintenance conditions (described above). hIPSCs were differentiated into endoderm, hindgut, and then intestinal organoids by using a previously published protocol (22). For differentiation into endoderm, the medium was changed to CDM-PVA supplemented with 100 ng ml^−1^ activin A (R&D Systems), 100 ng ml^−1^ bFGF (R&D Systems), 10 ng ml^−1^ bone morphogenetic protein 4 (BMP-4; R&D Systems), 10 μM phosphoinositol 3-kinase inhibitor LY294002 (Promega), and 3 μM CHIR99021 (Stratech Scientific Ltd.), which inhibits GSK3, resulting in WNT activation (days 2 and 3, day 3 without CHIR99021). To pattern the definitive endoderm to hindgut, the medium was changed to RPMI/B-27 medium consisting of RPMI 1640 medium plus GlutaMAX, 10 ml of 50× B27 supplement, 5 ml of nonessential amino acids (Gibco), and 10,000 U ml^−1^ penicillin-streptomycin (Invitrogen) supplemented with 100 ng ml^−1^ activin A and 100 ng ml^−1^ bFGF (days 4 and 5, day 5 without bFGF and with activin A at 50 ng ml^−1^). The medium was then changed to RPMI/B27 supplemented with 3 μM CHIR99021 and 3 μM retinoic acid (Sigma) (days 6 to 9). To generate three-dimensional (3D) intestinal organoids, hindgut tissue was washed with phosphate-buffered saline (PBS), incubated at 37°C with collagenase, and removed from the plate via gentle cell scraping. Tissue was manually broken up into smaller pieces, washed twice by centrifugation, and then resuspended in Matrigel (growth factor reduced phenol red free; Corning). Sixty-microliter droplets were added to each well of a 24-well plate, incubated for 15 to 20 min at 37°C, and then covered with 800 μl of iHO base growth medium: Advanced DMEM/F12 (Gibco), 10 ml of 50× B-27 serum-free supplement (Life Technologies), 5 ml of 100× N-2 supplement (Life Technologies), 1% 200 mM l-glutamine, 10,000 U ml^−1^ penicillin-streptomycin, supplemented with 500 ng ml^−1^ R-spondin 1 (R&D), 100 ng ml^−1^ Noggin (R&D), 100 ng ml^−1^ epidermal growth factor (EGF; R&D), 3 μM CHIR99021, 10 μM Y-27632 dihydrochloride monohydrate (Sigma), and 2.5 μM prostaglandin E_2_ (Sigma). The medium was changed every 2 to 3 days. iHOs were passaged every 4 to 6 days by dissolving Matrigel with Cell Recovery Solution (Corning), washing by centrifugation, manual disruption of the organoid ultrastructure via pipetting, and resuspension in fresh Matrigel overlaid with iHO base growth medium.

### Stimulation experiments.

After at least 6 weeks in culture A1ATD-1 iHOs were challenged with *S*. Typhimurium SL1344 (1 × 10^6^ CFU ml^−1^) or left uninfected. Stimulated organoids were incubated for 3 h at 37°C and 5% CO_2_, and then RNA was purified with the RNeasy minikit (Qiagen).

### Microinjection.

After at least 6 weeks in culture, iHOs were prepared for microinjection 4 days prior to infection by passaging as described above. Disaggregated iHOs were resuspended in 200 μl of Matrigel, plated into glass bottom Willco Wells microinjection dishes (INTRACEL), and covered with iHO base growth medium. The Eppendorf TransferMan NK2-FemtoJet express system was used to inject bacteria into organoids. All injections were carried out in a chamber at 37°C and 5% CO_2_. After injection, organoids were incubated for 3 h at 37°C and 5% CO_2_ and then fixed for microscopy. For RNA sequencing (RNA-Seq) analysis, 30 organoids per microinjection plate were injected with either PBS or *S*. Typhimurium SL1344 mixed 1:1 with phenol red dye. iHOs were incubated for 3 h at 37°C and 5% CO_2_, Matrigel was dissolved with Cell Recovery Solution (Corning), and phenol red-marked iHOs were isolated. RNA was then purified with the RNeasy minikit (Qiagen).

### RT-qPCR.

RNA isolated from whole iHOs before or after injection was reverse transcribed with the QuantiTect reverse transcription (RT) kit (Qiagen) according to the manufacturer's protocol. All RT-quantitative PCR (qPCR) experiments were performed with TaqMan gene expression assays and TaqMan gene expression master mix (Applied Biosystems) on the Applied Biosystems StepOne real-time PCR system. RT-qPCR data were analyzed via the comparative *C_T_* method with glyceraldehyde 3-phosphate dehydrogenase (GAPDH) as an endogenous control.

### RNA-Seq and analysis.

RNA was prepared from iHOs microinjected with *S*. Typhimurium SL1344 and iHOs microinjected with PBS (as described above) for three biological replicates per condition. Multiplexed mRNA libraries were prepared by using the Illumina TruSeq protocol and sequenced via paired-end sequencing with the Illumina-C HiSeq 2500 platform. Each lane of Illumina sequence was assessed for quality on the basis of GC content, average base quality, and adapter contamination. RNA-Seq reads were aligned with TopHat (23) version 2.0.8 with the human reference version GRCh37 used for the 1000 Genomes project. The read counts per gene were generated with featureCounts version 1.4.5-p1. The annotation for featureCounts came from ENSEMBL 75. Read counts were used to represent gene expression levels. R version 3.1.2 was used to import count data, and the DESeq2 package was used to normalize the data and detect differentially expressed genes (24). Significantly differentially expressed genes were selected with a cutoff false-discovery rate of less than 0.05 and a log change of >2.0-fold. Heat maps and principal-component analysis (PCA) plots were constructed from rlog-transformed data with the ggplot2 R library (http://cran.r-project.org/web/packages/ggplot2/index.html). For the 250 most significantly upregulated genes, enriched gene ontology terms were identified by using InnateDB with the category Biological Processes selected.

### Anti-human cytokine/chemokine multiplex bead assays.

Triplicate 25-μl samples of iHO culture supernatants from unstimulated iHOs and iHOs stimulated with *S*. Typhimurium SL1344 by addition to the culture medium were analyzed for cytokine/chemokine concentrations. Millipore customized anti-human Milliplex magnetic bead kits were used in accordance with the manufacturer's instruction, and a multiplex selection of the analytes tumor necrosis factor alpha (TNF-α), interleukin-6 (IL-6), and IL-8 was measured. Briefly, 25-μl aliquots of supernatants or dilutions of a mixed cytokine standard were captured on anticytokine antibody-coated beads overnight. On the following day, after washing with a 96-well plate-formatted magnet, detection reagents were added and allowed to bind to any captured cytokine present on the beads. Following further washes with the 96-well magnet, data were acquired on a Luminex FlexMap3D and analyzed with Luminex X-exponent software by extrapolation from the standard-curve dilutions. Results are expressed in picograms per milliliter.

### Immunohistochemistry.

For immunohistochemical analysis, iHOs suspended in Matrigel were fixed in 3% paraformaldehyde and 0.125% glutaraldehyde in PBS for 1 h at room temperature. They were then cooled to 4°C and dehydrated through an ethanol series (25, 50, 70, and 100% for 30 min each), infiltrated with glycol methacrylate (GMA) JB-4 and benzoyl peroxide (JB-4 embedding kit; Sigma-Aldrich), polymerized in gelatin capsules at 4°C for 24 to 48 h, and stored at −20°C. One- to 2-μm sections prepared on a Leica UCT ultramicrotome were mounted on slides precoated with 0.01% (wt/vol) poly-l-lysine hydrobromide. For immunoperoxidase staining, samples were blocked for 30 min with 0.1% sodium azide and 100 μl of 30% hydrogen peroxide and for 30 min with RPMI 1640 culture medium (Invitrogen) with 5% fetal calf serum and 1% BSA and then washed with TBS buffer (0.6 g of Tris, 8 g of sodium chloride, 1 liter of distilled H_2_O [dH_2_O], 3 to 4 ml of 1 M HCl to pH 7.6) three times for 15 min each. Samples were incubated overnight at 4°C with primary antibodies for mucin 2 (MUC2; ABCAM ab11197), lysozyme (LYZ; ABCAM ab2408), and chromogranin A (CHGA; ABCAM ab36997) in TBS buffer at antibody-dependent dilutions (MUC2, 1:200; LYZ, 1:50; CHGA, 1:50). Bound primary antibodies were detected with the EnVision+ System-HRP (AEC) (Dako), and nuclei were visualized by counterstaining with Mayer's hematoxylin. Finally, sections were covered with GeneTex Clear Mount (Sapphire Bioscience) and baked at 80°C for 5 min to set. For immunofluorescence staining for LAMP-1 (ABCAM ab24170), followed by donkey anti-rabbit 647 (ABCAM ab150075) and Salmonella common structural antigen 1 (CSA-1), fluorescein isothiocyanate-labeled (Insight Biotechnology Limited 02-91-99) sections were similarly processed by omitting the first block and diluting all of the antibodies in PBS (LAMP-1, 1:50; donkey anti-rabbit 647, 1:100; CSA-1, 1:20). Sections were mounted in Prolong-Gold with added 4′,6-diamidino-2-phenylindole (DAPI; Invitrogen).

### Transmission electron microscopic analysis of infected iHOs.

Infected organoids were fixed in 2.5% glutaraldehyde and 2% paraformaldehyde in 0.1 M sodium cacodylate buffer (1 liter of dH_2_O, 21.4 g of sodium cacodylate, 1 g of MgCl_2_, 0.5 g of CaCl_2_, adjusted to pH 7.42 with HCl), postfixed in 1% osmium tetroxide diluted in sodium cacodylate buffer, dehydrated with an ethanol series, and then embedded with the Epoxy Embedding Medium kit (Sigma-Aldrich). After embedding, samples were cured at 65°C for 48 h. Semithin (0.5-μm) sections were cut on a Leica UCT ultramicrotome and stained with toluidine blue on a microscope slide with suitable areas selected for ultrathin 50-nm sectioning. Ultrathin sections were collected on copper grids and contrasted with uranyl acetate and lead citrate before viewing on an FEI 120-kV Spirit BioTWIN transmission electron microscope. Images were taken on an F4.15 Tietz charge-coupled device camera.

### Invasion assays.

Microinjection was carried out as described above. To assess the invasion of iHO epithelial cells by bacteria, we modified the commonly used gentamicin protection assay (25) for use in iHOs. Forty iHOs per microinjection dish were injected with either wild-type or *invA* mutant *S*. Typhimurium SL1344. Injected iHOs were incubated for 90 min at 37°C and 5% CO_2_. iHOs were isolated from Matrigel with Cell Recovery Solution (Corning), centrifuged, washed once with PBS, subjected to manual disaggregation of the organoid ultrastructure, resuspended in iHO base growth medium supplemented with 0.1 mg ml^−1^ gentamicin (Sigma), and incubated at 37°C for 1 h. iHO aggregates were then centrifuged and washed once with PBS (Sigma). Cells were lysed with 1% Triton X-100 in PBS. Lysates were serially diluted in PBS, and 20-μl spots were plated onto prewarmed LB plates for CFU counting. Protection assays were performed with three biological replicates.

### Statistical analysis.

For statistical comparisons, we used unpaired, two-tailed *t* tests done with the Prism 6.0b software (GraphPad).

### Nucleotide sequence accession numbers.

RNA-Seq data are stored in the European Genome-Phenome Archive under study accession number EGAS00001001253. Data will be made available to all researchers upon request to the Data Access Committee (DAC) for the Wellcome Trust Sanger Institute, accession number EGAC00001000205. The named person of contact for the DAC for the Wellcome Trust Sanger Institute is Giselle Kerry (gh2@sanger.ac.uk). The restriction on data access is required for human donor protection.

## RESULTS

### IHOs generated from A1ATD-1 hIPSCs recapitulate features of the human intestine *in vitro*.

**FIG 1:**
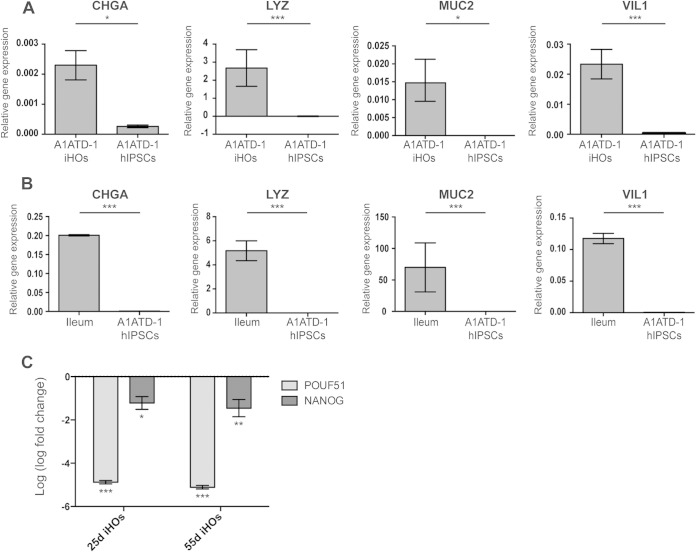
A1ATD-1 hIPSC-derived intestinal organoids show increased expression of genes for markers of adult intestinal tissue. (A) At differentiation day 30, the expression of adult intestinal markers *CHGA* (enteroendocrine cells), *LYZ* (Paneth cells), *MUC2* (goblet cells), and *VIL1* (enterocytes) was significantly greater in A1ATD-1 iHOs than in A1ATD-1 hIPSCs (*P* = 0.0023, *P* = 8.41 × 10^−5^, *P* = 0.002, and *P* = 4.4 × 10^−5^, respectively). (B) Primers were validated by measuring the expression of the same markers in RNA isolated from human ileum. (C) Log fold change in the expression of pluripotency markers. The expression of *NANOG* and *POUF51* was significantly lower in A1ATD-1 iHOs on both days 25 (25d) (*P* = 4.64 × 10^−5^ and *P* = 1.99 × 10^−3^) and 55 (55d) (*P* = 3.55 × 10^−5^ and *P* = 3.63 × 10^−4^) than in their A1ATD-1 hIPSC progenitors. Each RT-qPCR was performed with a TaqMan gene expression assay specific for each gene and analyzed via the comparative *C_T_* method with GAPDH as an endogenous control. *, *P* < 0.05; **, *P* < 0.001; ***, *P* < 0.0001.

**FIG 2:**
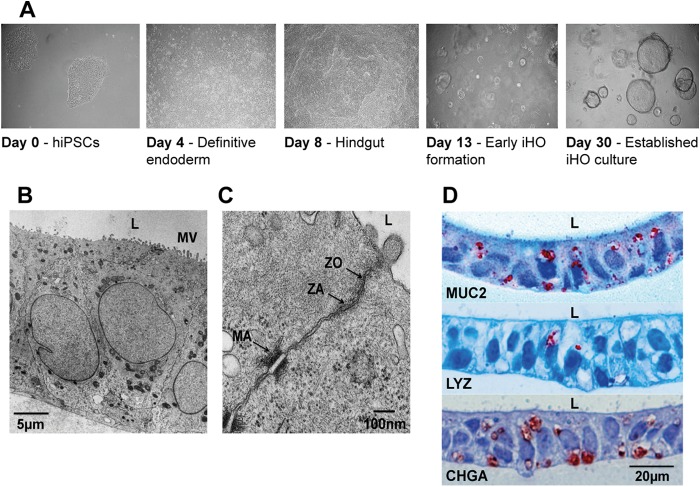
A1ATD-1 iHOs display a morphology characteristic of the human intestine. (A) Time course showing that directed differentiation of hIPSCs with defined concentrations of combinations of activin A, FGF, BMP-4, LY294002, and CHIR99021 results in the formation of a definitive endoderm at day 4. After 8 days, patterning of this definitive endoderm with defined concentrations of CHIR99021 and retinoic acid results in the formation of hindgut. Placement of this hindgut into a 3D intestinal culture system consisting of a supporting matrix of Matrigel and medium supplemented with prointestinal proliferation factors R-Spondin 1, Noggin, EGF, CHIR99021, and prostaglandin E2 results in the formation of small spheroids. Sustained maintenance and passaging of these spheroids result in the formation of established iHO cultures. Images were taken at ×100 magnification. (B) Transmission electron micrograph of established A1ATD-1 iHO wall demonstrates polarization of epithelial cells with a distinct luminal side (L) and microvilli (MV). (C) Transmission electron micrograph showing the presence of structures closely resembling tight junctions between cells (ZO, zonula occludens; ZA, zonula adherens; MA, maculae adherens). (D) Immunohistochemistry with marker-specific antibodies for mucin 2 (goblet cells), lysozyme (Paneth cells), and chromogranin A (enteroendocrine cells) validates the presence of intestinal secretory cell lineages in the iHO ultrastructure.

Human intestinal organoids were generated from A1ATD-1 hIPSCs, which were initially reprogrammed from dermal fibroblasts (21), in accordance with a previously published differentiation protocol (22). After 30 days in culture, there were significantly higher levels of the mRNAs for *MUC2* (mucin 2, goblet cells), *VIL1* (Villin 1, enterocytes), *CHGA* (chromogranin A, enteroendocrine cells), and *LYZ* (lysozyme, Paneth cells) in A1ATD-1 iHOs than in A1ATD-1 hIPSCs (Fig. 1A). These four markers were also highly expressed in control human ileal tissue (Fig. 1B). In contrast, 25 days after resuspension in Matrigel and the addition of iHO base growth medium, we observed dramatically lower mRNA levels of the pluripotency markers *NANOG* and *POUF51* in iHOs than in A1ATD-1 hIPSCs (Fig. 1C). We also observed distinct morphological changes in our hIPSC cultures marking the differentiation process to endoderm, hindgut, and finally iHOs (Fig. 2A). After 1 to 2 weeks in Matrigel, the iHOs formed spheroid structures of cells enclosing a hollow lumen, with some crypt-like structures evident in the iHO ultrastructure. To further dissect the morphology of the iHO wall, we examined iHO cellular structure in more detail by transmission electron microscopy (Fig. 2B). We were able to visualize a polarized monolayer of epithelial cells, with a clear distinction between the apical and basal sides, and microvilli present on the apical surface. Structures resembling tight junctions between epithelial cells were also evident (Fig. 2C). Finally, we detected the expression and localization of MUC2, LYZ, and CHGA in iHOs with marker-specific antibodies and immunohistochemistry within subsets of cells in the iHO wall (Fig. 2D). The cells expressing these markers were distributed around the iHO and were not solely congregated in one region, illustrating that A1ATD-1 iHOs comprise enterocytes interspersed with cells harboring genetic signatures typical of Paneth, goblet, and enteroendocrine cells.

### Stimulation of A1ATD-1 iHOs with *S*. Typhimurium SL1344 results in altered patterns of gene expression.

**FIG 3:**
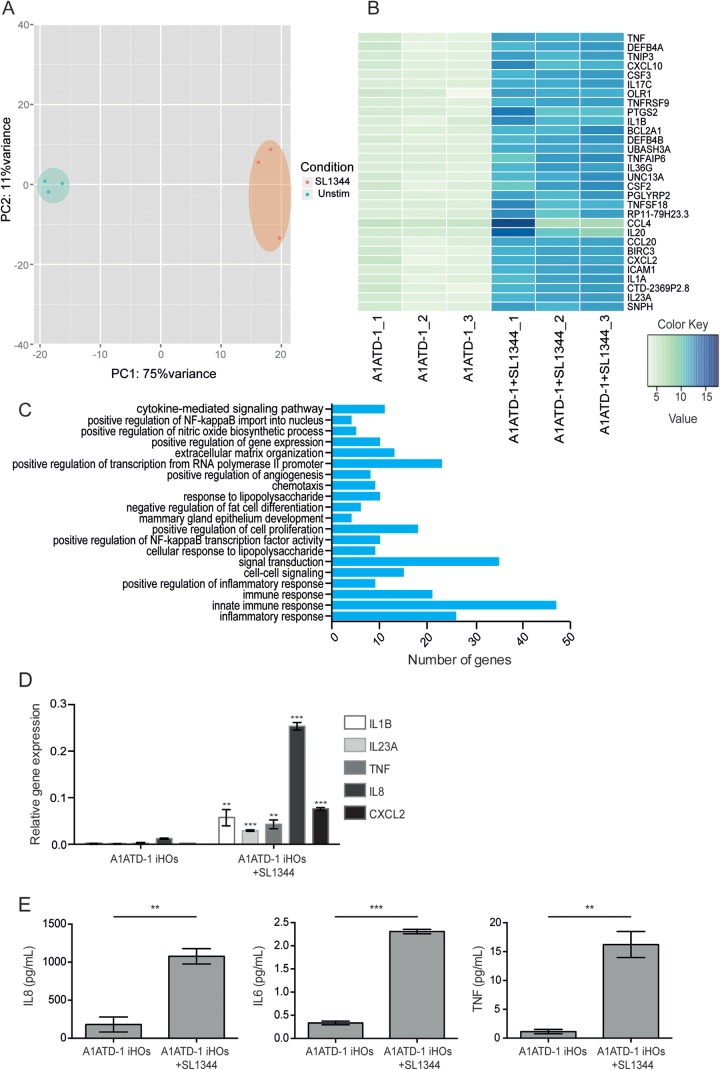
Microinjection of A1ATD-1 iHOs with *S*. Typhimurium SL1344 results in upregulation of genes associated with infection and inflammation. After at least 6 weeks in culture, A1ATD-1 iHOs were microinjected with a mixture of phenol red dye and *S*. Typhimurium SL1344 with the Eppendorf TransferMan NK2-FemtoJet express system. iHOs were incubated at 37°C and 5% CO_2_ for 3 h, phenol red-marked organoids were isolated, and RNA was prepared. (A) PCA of RNA-Seq expression data for three biological replicates per condition illustrates distinct differences in gene expression patterns between iHOs stimulated with SL1344 and unstimulated iHOs. (B) Heat map of the RNA-Seq expression data calculated with DESeq2 for the 30 most significantly upregulated genes after the addition of SL1344 to A1ATD-1 iHOs. (C) Enriched biological processes upregulated after stimulation of A1ATD-1 iHOs with *S*. Typhimurium SL1344 (for the genes associated with each pathway and *P* values, see Data Set S1 in the supplemental material). (D) RT-qPCR showing that transcripts for the cytokines IL-8, CXCL2, IL23A, TNF-α, and IL1B are significantly upregulated in iHOs injected with SL1344 in comparison to those in iHOs injected with PBS (*P* = 1.56 × 10^−9^, *P* = 2.35 × 10^−9^, *P* = 4.21 × 10^−8^, *P* = 1.28 × 10^−4^, and *P* = 6.42 × 10^−4^, respectively). RT-qPCR data were analyzed via the comparative *C_T_* method with GAPDH as an endogenous control. (E) After stimulation of iHOs with *S*. Typhimurium SL1344, induction of TNF-α, IL-6, and IL-8 production was shown to be significantly upregulated with Luminex cytokine assays of supernatants collected from stimulated and unstimulated iHOs (*P* = 3.34 × 10^−4^, *P* = 6.33 × 10^−7^, *P* = 3.89 × 10^−4^, respectively). Assays were performed with supernatants from three biological replicates.

To probe the changes in the transcriptome of A1ATD-1 iHOs induced after infection of iHOs with salmonellae, we microinjected iHOs with *S*. Typhimurium SL1344 or PBS as a control and performed a global RNA-Seq analysis. After differential expression analysis, we observed 1,448 genes significantly upregulated in iHOs infected with *S*. Typhimurium SL1344 (cutoff, log change of >2-fold, adjusted *P* value, <0.01) and 577 genes significantly downregulated (cutoff, log change of <2-fold; adjusted *P* value, <0.01) compared to controls. PCA (Fig. 3A) demonstrated clustering of the samples dependent on treatment, with those samples infected with SL1344 forming a group distinct from the uninjected iHOs, suggesting different patterns of gene expression in A1ATD-1 iHOs injected with SL1344. A heat map of the 30 most highly upregulated genes in iHOs after injection with *S*. Typhimurium SL1344 consists of a high proportion of genes associated with response to infection (Fig. 3B). ILs, essential mediators of the interactions between immune cells and nonhematopoietic cells (26), comprise 6 of the 30 most highly upregulated genes after infection of iHOs with SL1344. One of the highly expressed ILs was *IL-23A*, which has recently been linked with protection of the intestinal barrier through interaction with the IL-22 signaling pathway (27). *IL-20* is also highly expressed after infection with SL1344, and this IL is associated with epidermal function and psoriasis (28); however, its role in intestinal infection is undetermined (29). After differential-expression analysis, we detected significant upregulation of goblet cell-associated genes such as *GCNT3* and *MUC2* (30) (log_2_ change, 1.5022-fold; adjusted *P* value, 1.68 × 10^−46^; log_2_ change, 1.3399-fold; adjusted *P* value, 0.0059, respectively), suggesting that multiple cell types that make up the iHO can respond to SL1344 stimulation. Interestingly, one of the highly upregulated genes was *BIRC3*, which is also upregulated in enteroendocrine cells infected with Chlamydia trachomatis (31). Many genes encoding proinflammatory cytokines, including *CCL20*, *IL1B*, *IL23A*, *CSF1*, *CXCL10*, *IRAK2*, *TLR7*, *TNF*, *TNIP1*, *TNFAIP6*, and *CCL22*, were also significantly upregulated. Factors involved in the innate immune response, inflammation, cytokine-mediated signaling, and the response to lipopolysaccharide were all enriched in iHOs after infection with *S*. Typhimurium SL1344 (Fig. 3C; see Data Sets S1 and S2 in the supplemental material). To validate transcriptional changes in iHOs after infection with *S*. Typhimurium SL1344, we carried out further microinjections with PBS or SL1344 and measured the mRNAs for *IL-8*, *IL1B*, *IL23A*, *TNF*, and *CXCL2* with quantitative assays (Fig. 3D). The relative log fold expression of the mRNAs for these five genes increased significantly in iHOs infected with SL1344, in comparison with that in organoids infected with PBS. Because of the morphology of organoids in culture, it is possible to stimulate them apically via microinjection or basally through addition to the culture medium. We produced a global RNA-Seq data set by using basally stimulated iHOs. To this end, we left clusters of iHOs untreated or stimulated them with *S*. Typhimurium SL1344 by independent addition to the iHO base growth medium and performed an RNA-Seq analysis complementary to that carried out for the microinjected samples. Forty-nine of the 100 most highly upregulated genes were replicated in both data sets (see Data Set S1 and S3 in the supplemental material), and we observed enrichment in similar pathways after a gene ontology term analysis (see Data Sets S3 and S4 in the supplemental material). After stimulation with *S*. Typhimurium SL1344, we measured the expression of the cytokines TNF-α, IL-6, and IL-8 with Luminex cytokine assays to confirm their production and secretion by iHOs (Fig. 3E). We detected significantly greater expression of all three cytokines in the supernatants of SL1344-stimulated iHOs than in unstimulated iHOs (*P* = 3.34 × 10^−4^, *P* = 6.33 × 10^−7^, and *P* = 3.89 × 10^−4^, respectively).

### Microinjection of iHOs allows direct modeling of *S*. Typhimurium interaction with intestinal cells.

**FIG 4:**
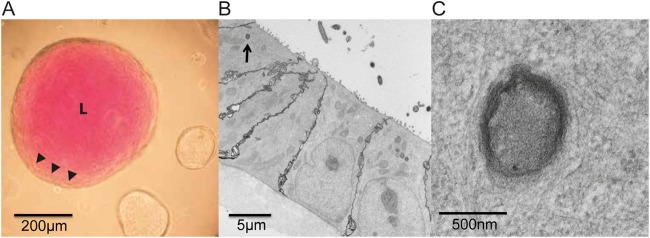
Microinjection of organoids derived from hIPSCs provides a model of *S*. Typhimurium SL1344 infection. Overnight cultures of *S*. Typhimurium SL1344 were diluted 1:1 with phenol red and injected into the lumen (L) of A1ATD-1 iHOs with the Eppendorf TransferMan NK2-FemtoJet express system. (A) iHOs retained the inoculum for 3 h subsequent to injection. Arrowheads mark the interface between the bacterial inoculum and human cells. (B and C) Transmission electron micrographs 3 h after injection showing SL1344 populating the lumen of the iHO and residing inside the epithelial cells in SCVs after invasion (arrow) (B; enlarged in panel C).

Our RNA-Seq analysis highlighted changes in the global transcriptional landscapes that are induced after pathogen sensing by the intestinal epithelial cells that make up the iHO structure. To further model bacterial invasion, we wanted to determine if the iHO system recapitulated hallmarks of Salmonella invasion such as the formation of the Salmonella-containing vacuole (SCV). Previous studies have used organoid dissection to introduce pathogens into the luminal cavity (12); however, we found that a microinjection system was a robust method (16). We could inject iHOs with a mixture of a bacterial suspension and phenol red, which made it easy to distinguish injected and noninjected iHOs in a single culture dish (Fig. 4A). After injection, iHOs retained their inoculum for the 3-h incubation time. We observed *S*. Typhimurium in the iHO lumen, close to the microvilli (Fig. 4B), and also inside the epithelial cells, residing in structures that resemble SCVs (Fig. 4C).

### An *S*. Typhimurium *invA* mutant is deficient in invasion of iHOs.

**FIG 5:**
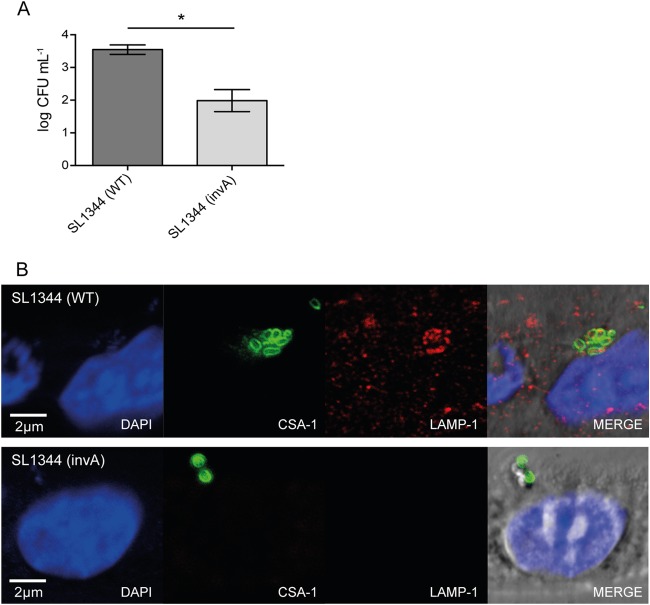
*S*. Typhimurium SL1344 *invA* mutant is deficient in the invasion of epithelial cells of iHOs. A1ATD-1 iHOs were microinjected with wild-type (WT) or *invA* mutant SL1344 and incubated at 37°C and 5% CO_2_ for 90 min. (A) Log numbers of CFU per milliliter recovered from cells of iHOs after microinjection and modified gentamicin protection assay, showing that WT SL1344 is significantly (∼30×) more invasive than the *invA* mutant (*P* = 0.0092). (B) Fluorescence staining of GMA JB-4- and benzoyl peroxide-fixed sections for CSA-1 (green) and LAMP-1 (red) and an overlay of the two images showing the colocalization (yellow) of bacteria and host protein after invasion by WT SL1344. *invA* mutant SL1344 was distinguishable from WT SL1344 by its lack of induction of LAMP-1 expression.

To further confirm the potential of the iHO system in studying Salmonella infection, we investigated the ability of an *S*. Typhimurium SL1344 *invA* mutant to infect following microinjection into the iHO lumen. InvA is an important component of the SPI-1 invasion system, and this derivative is attenuated in terms of the ability to enter epithelial monolayers (32). To address how this mutant behaved in the iHO system, we modified the gentamicin protection assay (25) regularly used to determine the percentage of bacterial invasion of epithelial monolayers. We found that after the microinjection of either wild-type or *invA* mutant *S*. Typhimurium SL1344, we recovered significantly different numbers of CFU after plating epithelial cell lysates (*P* = 0.0092), with SL1344 *invA* showing ∼30-fold less invasiveness (Fig. 5A). To confirm our finding, we fixed injected organoids after 2 h of incubation and stained them for host protein LAMP-1 and Salmonella CSA-1 (Fig. 5B). In iHOs infected with the *S*. Typhimurium *invA* mutant, we detected no salmonellae inside cells and an almost undetectable positive signal for LAMP-1, suggesting that the *invA* mutant was not being internalized and the host cell autophagy pathways were not being significantly triggered. In contrast, wild-type SL1344 could be found localized within vacuoles inside epithelial cells, frequently colocalized with LAMP-1, in a similar manner to that observed in other *in vivo* and *in vitro* systems (33).

## DISCUSSION

We show here that the iHO system is a novel and effective way to model aspects of the interaction of the enteric pathogen *S*. Typhimurium with the human intestinal epithelium. The iHOs that we generated from A1ATD-1 hIPSCs display important characteristics of the intestinal epithelium, such as clear polarization of epithelial cells and distinct cell types. We were able to observe large-scale transcriptional changes in A1ATD-1 iHOs in response to *S*. Typhimurium. Dissecting the role of the intestinal epithelium in defense against *S*. Typhimurium is important, as these cells are the entry points for *S*. Typhimurium into the underlying tissue and are therefore integral with the innate immune response (1). However, in whole-mouse infection models, epithelial interactions are difficult to analyze because of their rarity in the unligated intestine, and traditional immortalized intestinal cell lines lack the architecture and the mixture of absorptive and secretory cells that make up the intestinal epithelium *in vivo*. The combined action of different cell types may play a role in influencing the systemic spread of bacteria such as salmonellae, and the iHO system provides an opportunity to dissect the roles of these cell types further, particularly those that have proven difficult to culture *in vitro* via traditional cell culture techniques. Furthermore, the use of microinjection to introduce *S*. Typhimurium to the apical side of epithelial cells mirrors aspects of *S*. Typhimurium infection *in vivo*.

Previous studies have used mouse primary intestinal organoids to study various aspects of *S*. Typhimurium infection (11, 13). Although these mouse organoids are technically less challenging and less time-consuming to generate, they lack human genetic specificity. Therefore, iHOs could find utility for human-restricted pathogens. Data presented here and in previous reports by other groups (16, 34) suggest that the intestinal organoids derived from hIPSCs are more small intestine like in structure because of the presence of Paneth-like cells and the villus-like protrusions that they develop. *S*. Typhimurium invades at small intestinal sites in humans, making the iHO model appropriate for studies of this organism.

As we have shown here, the iHO system can be used to assess the pathogenesis of different bacterial mutants. iHOs also provide the opportunity to use information from human and bacterial genetic studies and rapidly apply that information in this tractable *in vitro* setting, allowing the effects of different genetic variants on the intestinal cellular phenotype to be established (35). For example, it is possible to generate hIPSCs, and subsequently iHOs, from individuals with distinct genetic architectures or those harboring mutations in known human genes (36). Since both pathogen and host variations play important roles in the establishment and subsequent development of Salmonella infections, having a system that enables the application of both could prove to be valuable.

## Supplementary Material

Supplemental material
