# Supplementary material for: Interaction of Salmonella enterica Serovar Typhimurium with Intestinal Organoids Derived from Human Induced Pluripotent Stem Cells
Source: Infect Immun. 2015 Jun 15;83(7):2926–34. doi: 10.1128/IAI.00161-15 (PMC4468523; doi:10.1128/IAI.00161-15)
Supplement: Supplemental material [file supp_83_7_2926__index.html]

Interaction of Salmonella enterica Serovar Typhimurium with Intestinal Organoids Derived from Human Induced Pluripotent Stem Cells — Supplemental material 

# Interaction of Salmonella enterica Serovar Typhimurium with Intestinal Organoids Derived from Human Induced Pluripotent Stem Cells

## Supplemental material

- Supplemental file 1 -

  Dataset S1. Top 100 genes significantly upregulated after microinjection of *Salmonella* Typhimurium SL1344 into iHOs, accompanied by gene symbols, gene names, significance *P* values, and raw counts in stimulated and unstimulated iHOs. Dataset S2. Top 50 biological processes identified using innate DB enriched in A1ATD-1 iHOs after microinjection of A1ATD-1 iHOs with *S.* Typhimurium SL1344. Dataset S3. Top 100 genes significantly upregulated after stimulation by *Salmonella* Typhimurium SL1344 in iHOs, accompanied by gene symbols, gene names, significance *P* values, and raw counts in stimulated and unstimulated iHOs. Dataset S4. Top 50 biological processes identified using innate DB enriched in A1ATD-1 iHOs after stimulation of A1ATD-1 iHOs with *S.* Typhimurium SL1344.

  XLSX, 41K
